# An Explanation of Shortening Heat Generation and Mechanical Performance Enhancement during Muscle Stretch

**DOI:** 10.1100/tsw.2001.102

**Published:** 2001-10-12

**Authors:** Dan Eremia

**Affiliations:** Department of Biophysics, “Carol Davila” University of Medicine and Pharmaceutics, Bdul Eroilor Sanitari nr. 8, 76241 Bucharest, Romania

**Keywords:** cross-bridges theory, N-filament, titin, titin modular structure, sarcomere, shortening heat, enhancement of muscle mechanical performance

## Abstract

Titin is an extraordinary long multidomain protein spanning half of the sarcomeric unit. One end of the titin filament is known to emerge from the Z-line, but the precise localization of the other end is still a matter of debate. Assuming that titin filaments connect the Z-line to the tips of thin filaments in the opposite half of the sarcomere, the behavior of an actively contracting muscle during mechanical stretch, as well as the mechanism of muscle shortening heat generation, can be easily explained if one considers that titin filaments are involved not only in the passive elasticity of the muscle but also in the force and heat generation during contraction.

## N-FILAMENTS AND THEIR POSSIBLE INVOLVEMENT IN MUSCLE FORCE AND HEAT GENERATION

It is obvious that the contractile machinery of the sarcomere cannot work properly in the absence of some filamentous system of longitudinal superthin structures in the sarcomere (besides thin and thick filaments and possibly interconnecting with them). The so-called “power stroke” of the cross-bridges (according to the cross-bridges theory) could be effective only if the thin filaments were entirely stiff or sufficiently stretched in order to be kept permanently rectilinear. Otherwise, strange as it may seem, the change of the angle between myosin head and actin monomer would produce the bent of the thin filament in the plane of the myosin head and the thin filaments’ axis, rather than the pull of the thick filaments towards the Z-line. If two cross-bridges emerging from diametrically opposed thick filaments attach at the same axial level to a thin filament and work simultaneously (an improbable situation), the most probable result would again be the bent of the thin filament in a plane of the myosin heads and the thin filaments’ axes (perpendicular to the initial plane). The thick filaments would be pulled toward the Z-line only if three or more crossbridges emerging from thick filaments situated around the thin one and working synchronously attach at the same level to that thin filament (a practical impossible event).

It is also known that a muscle fiber can be experimentally stretched so far that the overlap of filaments disappears and a gap occurs between the ends of thick and thin filaments. However, after cessation of the stretch, muscle fibers are able to regain their initial length, together with their contractile properties[1]. When the muscle is reshortened, thin filaments do not bend (or fold) on coming in contact with the lattice of thick filaments; they penetrate it, occupy their proper positions, and interdigitate in the same highly ordered manner as before the stretch.

Undoubtedly, because of their great flexibility, the thin filaments must be guided in sarcomere by “ropes” or “threads” in order to be permanently stretched and kept rectilinear. The necessary longitudinal system of superthin filaments could have the four principal variants shown in Fig. 1.

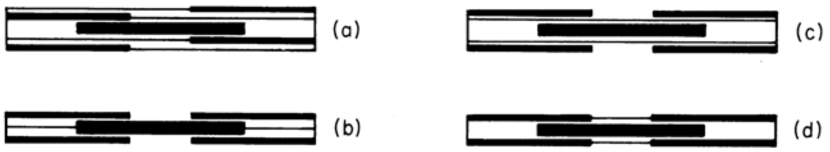

**FIGURE 1.** Graphical representation of the most probable continuous structures in sarcomere: (a) thin filament–thin filament structure; (b) Z–Z structure; (c) Z–thick filament structure; (d) Z–thin filament structure.

The structures connecting the terminals of thin filaments were identified in 1954 as S–filaments by Huxley and Hanson; the structures connecting the two Z-lines were identified in 1968 by Hoyle as T-filaments; and the structures connecting the Z-line to the ends of thick filaments were identified in 1977 by Ulrick as superthin filaments. The existence of filaments connecting the free ends of the thin filaments to the Z-line in the opposite half of the sarcomere (N-filaments) was, for the first time, proposed by us[2] as the only possible explanation of the strange behavior of the N-line in the tortoise muscle during the change in sarcomere length.

The N-filament was supposed to be made up by serially linked modules. If these modules were able to fold, thus participating in the force and heat generation in muscle (Fig. 2), a large body of knowledge of muscle contraction could be readily assimilated into this framework of ideas, assuming also that the module folding is an exothermic process.

## TITIN AS A PERFECT CANDIDATE FOR THE N-FILAMENT STRUCTURE

Titin is known as an extraordinary long multidomain protein, spanning half of the sarcomeric unit from the Z-disc to the M-line. One end of the titin filament is known to emerge from the Z-line of the sarcomere; the precise localization of the other end is still a matter of debate. In our view, titin filaments are supposed to span from Z-line to the tips of thin filaments in the opposite half of the sarcomere.

In a sarcomere at resting length, where the H-zone is very narrow, tips of thin filaments emerging from the two opposite ends of the sarcomere are positioned practically in the middle of the sarcomere, so that titin filaments indeed span only half of the sarcomeric unit. Many laboratories, however, have demonstrated that epitopes of the titin filaments in the A-band and A-I junction regions do not change their position relative to the thick filaments during muscle shortening and stretching. Surprisingly enough, this fact does not contradict our view. As you can see in Fig. 3, central domains of the N-filament always stay close to neighboring thick filaments, no matter the length of the sarcomere.

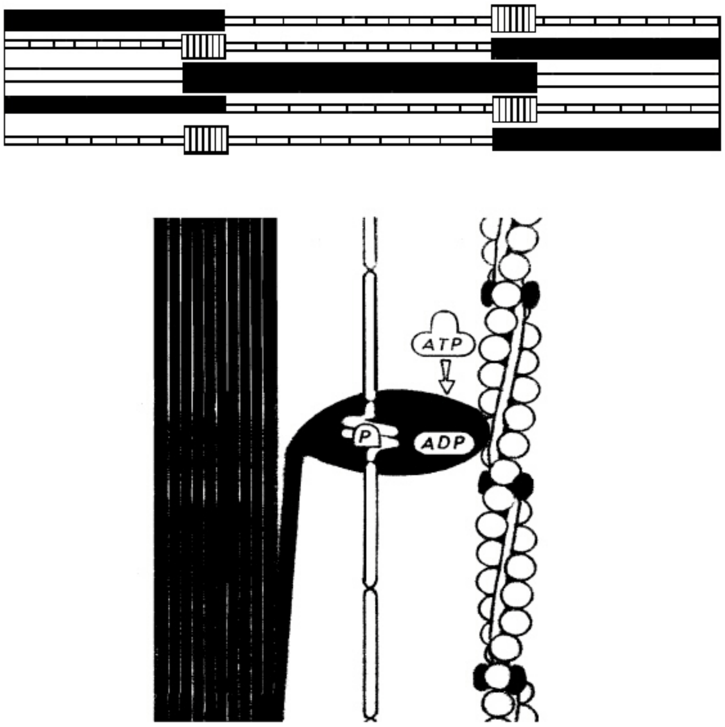

**FIGURE 2.** Schematic representation of the proposed N-filaments[2] made by serially linked modules able to fold as a consequence of ATP splitting in the overlap region of the thin and thick filaments.

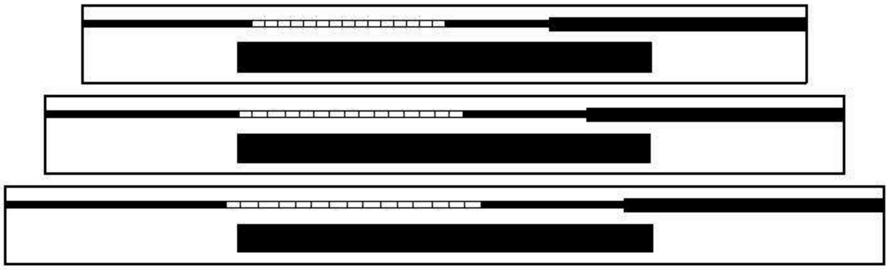

**FIGURE 3.** Schematic representation of the elastic N-filaments' behavior in sarcomere during stretching; the central domains (white boxes) of N-filament keep approximately the same rapports with their neighboring thick filaments.

Consequently, titin filaments seem to be a perfect candidate for the N-filaments we proposed in 1985[2].

The primary structure of titin consists of repeats of two types of approximately 100 amino acid modules (fibronectin type III and immunoglobulin-like motifs, respectively) and a Pro rich segment named PEVK[3]. Titin poly-immunoglobulin region is composed of 244 modules that may exist in either folded or unfolded conformation. The length of a folded module is 4.4 nm; the full unfolding of the module (observed under a 200 pN force) produces a 28.4 nm extension[4].

When a single titin molecule is experimentally subjected *in vitro* to stretching forces, its elongation features suggest that these forces cause consecutive, one-by-one unfolding of each domain in all-or-none fashion, with the amount of force needed for each module unfolding about 210 ± 27 pN for I 27 module and 264 ± 49 pN for I 28 module[4].

## TITIN MODULE FOLDING PROCESS AS A POSSIBLE SOURCE OF ACTIVE MUSCLE CONTRACTION FORCE AND OF MUSCLE SHORTENING HEAT

Currently, titin filaments are considered responsible only for the passive elasticity of the muscle. However, it is worthwhile to consider the possibility that titin modules actively fold in the muscle contraction as a consequence of ATP splitting in the overlap region of the thin and thick filaments, thus being able to participate in the contraction process by developing mechanical tension.

It is known[5] that the maximal force generated by a muscle with a transverse section area of 1 $cm^2$ is about 40 N; it is also known that this section of muscle contains about $2 \times 10^{11}$ thin filaments and, presumably, an equal number of N-filaments (supposedly made up of titin). If, as our hypothesis suggests, titin modules situated in the region of thin and thick filaments overlap actively fold during contraction, the force produced by such folding of a titin module may be easily calculated:

$$P = 40 \times 10^{12} \text{ pN} / 2 \times 10^{11} = 200 \text{ pN}$$

This value is very close to the amount of force necessary to mechanically unfold one folded titin module.

Shortening heat is defined as the total amount of heat produced in isotonic contraction, in excess of that produced in the isometric contraction at the same length of the sarcomere. It is known that shortening heat is proportional to the distance shortened, with a constant of proportionality approximately independent of the speed of shortening[6]. In all probability, this shortening heat cannot result from a greater rate of cross-bridges turnover[7], since it is not produced by ATP or phosphoryl-creatine splitting[8]. The shortening heat seems to be produced by an extra chemical reaction occurring in a shortening muscle, presumably a noncyclic reaction whose extent depends only on the extent of shortening[9].

The mechanism of the shortening heat production can be easily accounted for in terms of the model of N-filaments (titin filaments) as force generators in striated muscle, assuming that the folding of the titin module is an exothermic process. When the muscle is allowed to shorten, new unfolded titin modules will be continually introduced in the overlap and will fold, thus producing heat in excess as compared to the isometric contraction.

It is a known fact that there is no shortening heat at all if the amount of release to a slightly shorter length is less than 1% of the resting muscle length (about 10 nm per half sarcomere). Similarly, in bigger releases there is no shortening heat for the first 10 nm of shortening[10]. This distance of shortening is practically equal to the distance necessary for an unfolded titin module to enter the overlap region in order to fold and to liberate the shortening heat. This approach of muscle contraction satisfactorily explains the fact that shortening induces a temporal separation between the processes that liberate enthalpy and those that hydrolyze ATP[11].

The proposed model reveals a reasonable agreement with some values characteristic to skeletal muscle. The amount of the shortening heat for 1 cm of shortening of a muscle with a transverse section equal to 1 $cm^2$ is 34 mJ (8.5 mcal)[12]. If the shortening heat would be liberated by the titin module folding process, the corresponding heat for each module that folds will be:

$$8.5 \times 10^{-3} \text{ cal} \times 10^{-6} \text{ cm} / 2 \times 10^{11} \text{ titin modules} = 4.25 \times 10^{-20} \text{ cal}$$

For 1 mol, the heat will be:

$$4.25 \times 10^{-20} \times 6.23 \times 10^{23} = 26.4 \text{ kcal/mol}$$

Since the work required to rupture a pair of hydrogen bonds is about 10 kcal/mol, at least two pairs of hydrogen bonds seem to be formed in the folding of a titin module.

## TITIN MODULE UNFOLDING PROCESS MAY EXPLAIN THE BEHAVIOR OF THE CONTRACTING MUSCLE DURING MECHANICAL STRETCH

When an actively contracting skeletal muscle is forcibly lengthened, the mechanical tension developed by the muscle increases significantly during the stretch[13] (see Fig. 4). The plateau value of the force during stretch is independent of the amplitude of the stretch, but depends upon the velocity of stretch. In other words, the faster the muscle is stretched, the higher the recorded force. The recorded force is not proportional to the overlap of the thin and thick filaments and occurs without a corresponding increase in ATP splitting[14].

The force developed by muscle after stretch is independent of the stretch velocity and has practically the same value over a wide range of sarcomere length. The enhancement of mechanical performance by stretch during tetanic contraction of vertebrate skeletal muscle fibers has not yet received a satisfactory explanation.

On the other hand, the enthalpy produced by an actively contracting skeletal muscle is substantially reduced or altogether suppressed during the stretch[15]. There are two possible explanations for this reduction of the enthalpy: (1) an exothermic process is prevented by stretch or (2) an endothermic process is caused by stretch[16]. The reduction of enthalpy cannot be due simply to prevention of the exothermic ATP splitting[15]. Some endothermic process apparently must occur, but this process is neither ATP synthesis (because this was not found in a chemical experiment with a similar design[17]) nor heat absorption due to the thermoelastic effect (because it is much smaller[10]). This unknown endothermic process could be the reverse of the process responsible for the shortening heat.

According to our proposed model of muscle contraction, the beginning of the plateau of the forces recorded as response to stretch may indicate the very moment when the first folded titin module suddenly unfolds as the stretch has exceeded the critical value P (about 200 pN). After the first titin module “gives,” the tension tends to decrease, but the maintained stretch restores again the tension P and a new folded titin module will suddenly unfold and so on, until the stretch ceases. After the last titin module unfolds, the tension falls abruptly.

The small tension oscillations during the plateau (see Fig. 4) may, therefore, reflect successive unfolding processes of the folded titin modules. This general picture is consistent with some recent *in vitro* studies of the force-extension curve of a native titin fragment showing a characteristic sawtooth pattern[18].

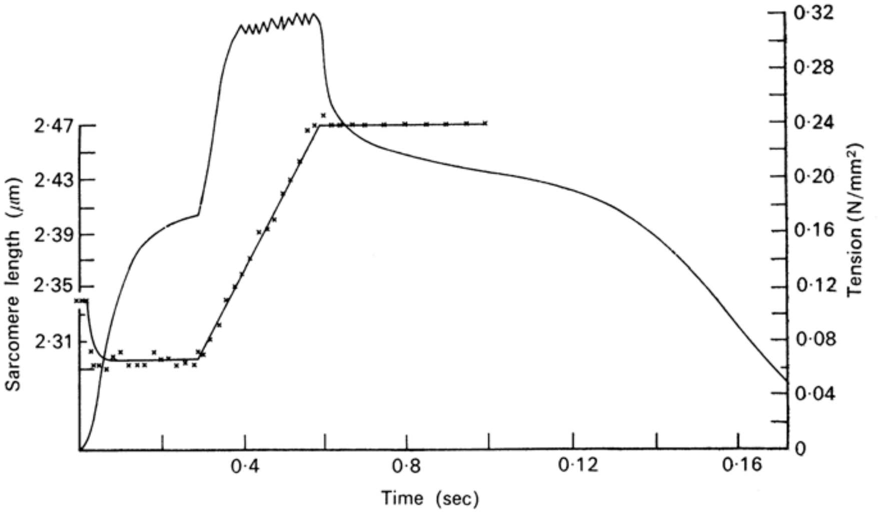

**FIGURE 4.** Effect of stretch on tension and sarcomere length during a tetanus. Measurements of sarcomere length (crosses) are superimposed on traced tension record (after Edman et al.)[13].

The fact that the plateau value of the muscle force during stretch, though independent of the amplitude of stretch, depends upon the velocity of stretch, is not surprising, because the unfolding of the folded titin module needs some time to occur and, if the stretch is too fast, the folded titin module will be temporarily overextended before eventually unfolding, thus generating a tension greater than P. What about the known fact that the magnitude of the force after stretch is independent of the stretch velocity and has practically the same value over a wide range of sarcomere length[13]? This is an expected feature if unfolding of the folded titin modules and refolding of the unfolded titin modules are considered to be in equilibrium under a tension normally reached in a short isometric tetanus for a sarcomere length between 1.9 and 2.3 μm (but reached only after the tension "creep" in a long tetanus if the sarcomere length exceeds 2.3 μm).

According to the molecular mechanism of muscle contraction that we suggest, the rate of titin module folding must be greater in the first part of an isometric tetanus as the unfolded titin modules already exist in the overlap fold, thus pulling in the overlap new unfolded titin modules which will fold in turn, the tension gradually rising until reaching a plateau.

## EVIDENCE FOR THE VALIDITY OF THE PROPOSED MODEL AS COMPARED WITH THE CLASSICAL CROSS-BRIDGES MODEL

The model of muscle contraction proposed in this paper can explain: (1) why the maximal speed of unloaded shortening (assumed in our opinion to reflect the speed of titin module folding itself) has a constant value independent of overlap; (2) why, like all natural and artificial contractile polymers, the muscle obeys Hill's hyperbolic dependence of the shortening velocity on the amount of the load lifted[19]; and (3) why myosin-free fibers contract when myosin $S_1$ is added[20]. The model can also account for many other aspects of the muscle contraction, including the so-called "catch" which is characterized by the presence of tension (or resistance to stretch) in the absence of the active state.

It is necessary to emphasize that not only does the cross-bridges theory fail to explain the above-mentioned phenomena, but that there are also many other disadvantages of the cross-bridges theory. One of the major drawbacks of the cross-bridges theory of muscle contraction is that it suggests that cross-bridges are supposed to be involved in mechanical oarlike cycles, with each mechanical step being associated with a distinct biochemical step.

The cross-bridges theory claims that the sliding process of myofilaments is driven by myosin cross-bridges that extend from the thick filaments and cyclically interact with the thin filaments, as adenosine triphosphate (ATP) is hydrolyzed. According to the Lymn-Taylor cross-bridge model (see the review of White and Thorson[21]), the myosin cross-bridge, complexed with ADP and $P_i$, successively binds actin filament with a rate constant of 100 $s^{-1}$, releases the products of ATP hydrolysis with a rate constant of 10 to 30 $s^{-1}$ in solution and 1.5 $s^{-1}$ in muscle fiber, binds a molecule of ATP at a very fast rate, detaches from the actin filament (with a rate constant 1000 $s^{-1}$), and hydrolyzes the bound ATP (with a rate constant 150 $s^{-1}$) so that the overall actomyosin ATP-ase cycle is as long as 50 to 125 ms in solution and about 700 ms in muscle fiber.

A comparative analysis of the actomyosin ATP-ase cycle and of the mechanical oarlike cycle of the cross-bridges raises some intriguing questions. The cross-bridge oarlike cycle is assumed to start with the attachment of the cross-bridge to the thin filament at an angle of 90º, followed by the "power stroke" (whereby the angle changes from 90 to 45º), by the detachment from the thin filament, and the reorientation to the initial 90º angle relative to thin filament.

According to the cross-bridges model, there is only one power stroke of the cross-bridge during an actomyosine ATP-ase cycle, and, because the length of the myosin head is 15 nm, two successive attachment points to the actin filament of an individual cross-bridge are a maximum of 11 nm apart. However, it is known that the maximum rate of the relative sliding of filaments in intact muscle under zero load is 6 $\mu m \times s^{-1}$, so that the time available for a cross-bridge to perform its mechanical cycle is no longer than 2 ms, i.e., 25 to 350 times less (depending mainly on the rate constant of the products-release step in muscle fiber) than the time needed for a myosin head to go through a complete ATP-ase cycle.

More acceptable than the classical cross-bridges theory seems to be the hypothesis of jet propulsion[22], according to which the observed movements in actin-myosin systems (at least *in vitro*) result from the rapid ejection of the Mg-ATP splitting products. In 1985, using myosin-coated polystirene beads and an oriented array of actin filaments, Spudich et al.[23] demonstrated for the first time that actin alone is sufficient to support movement of myosin at rates consistent with the speeds of muscle contraction. However, the driving force for the relative movement of actin and myosin filaments *in vitro* is much smaller then the similar force in skeletal muscle[5], so practically the entire force in muscle is generated, according our hypothesis, by the folding process of the titin modules as a result of ATP splitting in the thin and thick filaments overlap region. However, it is not impossible that jet propulsion forces may play some minor role in sarcomere contraction, especially in the events in the extreme shortening of the muscle where, certainly, something other than conventional sliding must occur in order to explain the abrupt decrease of the forces in the left side of the classical length-tension relation.
